# Supplementary material for: Chromothripsis during telomere crisis is independent of NHEJ, and consistent with a replicative origin
Source: Genome Res. 2019 May;29(5):737–49. doi: 10.1101/gr.240705.118 (PMC6499312; doi:10.1101/gr.240705.118)
Supplement: Supplemental Material [file supp_gr.240705.118_Supplemental_file_1.zip › contigs/annotated_contigs/DB57/contig.2.DB57_length_645_mean_cov_12.7813953488.docx]

**DB57_length_645_mean_cov_12.7813953488**

```
TCCTACTGGGAGGCCACCAGCCCCTGATGGACCAAGGGGGGTCTGAGGACCCCAAGGCATTGCACATAAGTGTTTGCATGCATACACAC
 >chr2:10485979-10486296 - E=5e-176
ACACACACAGCCTGGCTCTCTCACACATACACAGACACACACACCCCCAGGGCACACACTCAGACACACCCACACACTCACACTTCCAC

TTATTCCCACAATTGTTCTTCCTGGTTTGGATCAACAGACCATCTGTGTGCTTGTGTGACTTAGGAAATCTTCCAAAACACGGCAAAAG

TTCGCAGTCCTTGCCCCTTTTGTGGTGGGGGAGGGGCGTCATAAATAGTT|CCCCACCTCCCCCAGCCCTTCGTTCATGTCAGGCCTTA
                                                    >chr2:10532660-10532988 - E=1e-185
GGCCCCTCTCCGAGTCTCTGGAGAATGGCAGAGGGAGGTGGCAAGGCCAGGGGCTGCATGGCTGGACTGTCTGGAATCAAAGCCCAGCT

GGACTGTTTCCTATTTCCTAACAGTGTGACCTTGACCTGTTATCTCACCTCTCCCTGCTTCCACTCCCGCATTTGTAAAATGGAGATGA

CAATAGTACTACCTAGCTCATCAAGTTTTTGTAAAGATTAAATGAGCTAATGCACAACAAATGCTTGGTACATAGAAAGAACTCAATAA

ATGTCAACTATGACTGAAAGAGC
```
